# Carrying the burden of Obstetric fistula, no longer

**DOI:** 10.1016/j.eclinm.2019.05.005

**Published:** 2019-05-29

**Figure:**
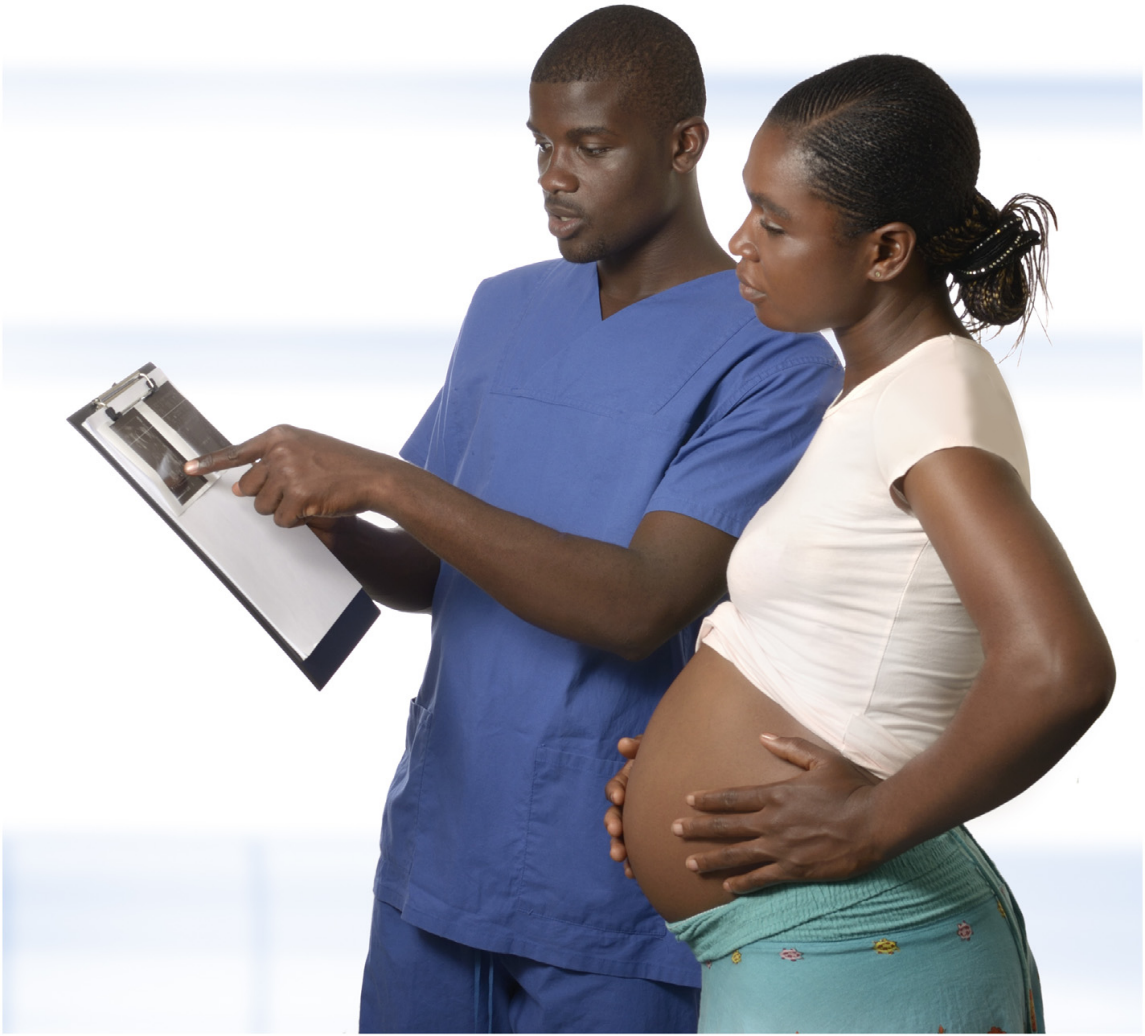

The ability simply to provide treatment to at risk populations is often underappreciated. For many maladies, the knowledge of how best to treat and prevent occurrence is hampered by the lack of access to care. The UN declares May 23^rd^ as “UN International Day to end Obstetric Fistula” and on the anniversary of this, we discuss the challenges and approaches to tackling this issue.

Obstetric fistula (OF) describes a devastating injury between the vagina and rectum or bladder that is caused by prolonged obstructed labour. In these instances, Childbirth may have proceeded for up to 7 days. The abnormal timespan of the child in the birth canals combined with contractions pushing the baby’s head against the pelvic bone, affects blood flow to these delicate tissues, resulting in fissures. As such, women who suffer from OF experience urine and/or faecal incontinence. Importantly, beyond the clinical characterisation of the injury, the negative social impact can be deeply burdensome. Women are ostracised in public, in their communities, in school, or at work, negatively impacting quality of life. In 2004 the global burden surrounding this condition was well known, leading Dr France Donnay and Nurse midwife, Laura Weil to write “The very existence of this condition is the result of gross societal and institutional neglect of women that is, by any standard, an issue of rights and equity.” In their comment to The Lancet. Sadly, the problem has not improved much since then.

OF affects an estimated 2 million women living in sub-Saharan Africa, Asia, the Arab region, Latin America and the Caribbean. The condition is also responsible for 6% of maternal deaths, causing devastating familial disruption, in these communities. Most shockingly, 50-100 thousand women each year continue to develop the injury. This estimated incidence is based purely on the number of women who seek treatment excluding women who decided not to or are unable to do so. More up to date information on the incidence and number of centres able to treat the condition is urgently needed.

OF is a healthcare concern born of the “haves and have nots” and more specifically “access”. In the global North, the incidence of OF is so low as to barely exist. This is markedly different to the prospects women in other parts of the world face. Challenges come in many forms. Reconstructive surgery is the most common treatment for OF and is successful in over 90% of cases. However, the specialist care and knowledge required of surgeons to treat OF successfully is scarce in the most at need areas. Niger, a country of over 20 million people with one of the highest birth rates in the world currently has only three medical centres capable of treating fistula patients.

In regions where, unqualified doctors are attempting fistula repair, there is an increase in women reporting post-operative issues. These women are caught between inadequately trained physicians and health centres with no options for treatment. Even if a woman with OF can source knowledgeable treatment, the estimated cost of surgery, post-operative care and rehabilitation support, at around $300 per patient is an additional hill to climb.

In the global South OF is a condition shouldered most often by young girls. Early marriage practices directly lead to increased cases of OF. Poverty and lack of resources can often result in for girls often younger than 15 to be wedded to males of varying ages. This practice occurs because marrying away children decreases the financial burden on the family and potentially reaps a dowry. Consequently, the age of marriage coincides with the first sexual experience for these children. The combination of malnutrition, poor education and undeveloped pelvises can result in OF. This societal practise seeks directly to impinge upon the wellbeing and health of millions of women in the global South and as a result accounts for the new cases adding to the burden of this injury, globally.

The resolution to end OF is part of the UN’s Sustainable development goals under “Good health and wellbeing”. This program declares that OF can largely be prevented by delaying the age of first pregnancy, the cessation of harmful traditional practices and timely access to obstetric care. The resolution also calls on the international community to raise awareness. Alongside, the UNFPA has organised workshops bringing together surgical expertise and governmental healthcare experts to discuss the way forward in specific regions.

So, what is the current status of OF globally? With the current availability of resources, it is estimated that it would take up to 400 years to treat the total backlog of women who suffer from the condition. Current estimates suggest that to begin fully preventing new cases of OF from occurring, there would need to be 75,000 new emergency obstetric centres built just in Africa. The Global fistula map provides useful information on trends but more complete and up to date information is required in order to better globally coordinate efforts.

Just this month, the first lady of Kenya, Margaret Kenyatta, launched the first National Strategic Framework for Prevention & Management of Obstetric Fistula. This practical approach seeks to provide knowledge to caregivers working on the frontlines of obstetric care. However, the true solution to this problem would require a more unified holistic approach to women’s health in the global South. A holistic approach not only describes treating women’s bodies but provides mental support for those who sustain the injury. Monetary investment from local health ministries combined with expertise from the global north in training local physicians is essential.

The path to eliminating OF is clear and well understood, therefore the only question remaining is whether the global community will take up the challenge.

*EClinicalMedicine*

